# Autophagy-mediated HMGB1 release promotes gastric cancer cell survival via RAGE activation of extracellular signal-regulated kinases 1/2

**DOI:** 10.3892/or.2015.3782

**Published:** 2015-02-04

**Authors:** QIU-YU ZHANG, LIN-QING WU, TAO ZHANG, YAN-FEI HAN, XU LIN

**Affiliations:** 1 Department of Immunology, School of Basic Medical Sciences, Fujian Medical University, Fuzhou, Fujian 350108, P.R. China; 2 Key Laboratory of the Ministry of Education for Gastrointestinal Cancer, Fujian Medical University, Fuzhou, Fujian 350108, P.R. China

**Keywords:** gastric cancer, HMGB1, autophagy, RAGE, ERK, MAPK

## Abstract

High mobility group box-B1 (HMGB1), an autophagy activator, is crucial in tumorigenesis. However, its extracellular role and signaling in gastric cancer remain unclear. Samples were collected from gastric cancer patients and healthy controls. Immunohistochemistry and immunocytochemistry were used to determine the localization of HMGB1 in gastric cancer tissues, four gastric carcinoma cell lines (BGC-823, SGC-7901, MKN-28 and MKN-45) and a gastric epithelial cell line GES-1. Western blot analysis and ELISA were used to assess the effects of gefitinib, an epidermal growth factor receptor inhibitor, on autophagy and HMGB1 release in BGC-823 cells. MTT assay and western blot analysis assessed the effects of extracellular HMGB1 on cell proliferation and signaling transduction. Released HMGB1 promoted proliferation through activation of ERK1/2 MAPK. HMGB1 expression in gastric cancer tissues and serum was significantly increased compared to the controls and healthy serum. Gastric carcinoma cells showed an increased HMGB1 in the nuclei and cytoplasm, whereas GES-1 cells exhibited a lower HMGB1 with nuclear localization. Gefitinib increased autophagy and cytoplasmic HMGB1 release from the BGC-823 cells. Extracellular HMGB1 in autophagic cell supernatant promoted proliferation that was abolished by glycyrrhizic acid, an HMGB1 inhibitor. BGC-823 cells incubated with HMGB1 had increased ERK1/2 phosphorylation, while levels of JNK, p38 or AKT were not affected. Blocking RAGE-HMGB1 interaction with antibody or siRNA suppressed the ERK1/2 activation and gastric cancer cell growth, indicating that RAGE-mediated ERK1/2 signaling was necessary for tumor progression.

## Introduction

High mobility group box-1 (HMGB1) is observed in most tumor types and its expression is higher in gastric adenocarcinomas (1–3). HMGB1 overexpression is associated with hallmarks of cancer, including unlimited replicative potential, vasculogenesis, evasion of apoptosis and insensitivity to growth inhibitors (4,5). HMGB1 was first identified as a non-histone chromosomal protein that participates in DNA replication, transcription and repair in most eukaryotic cells (6). Studies of the previous decade have reported that it also functions as an extracellular damage-associated molecular patterning molecule, promoting inflammation, cellular differentiation, survival and migration. Extracellular localization of HMGB1 may be mediated by passive release from necrotic cells or active secretion by inflammatory cells (7,8). Recent data show that certain cytotoxic chemotherapy agents may induce HMGB1 release that contributes to autophagy regulation (9,10).

Autophagy is a lysosome-mediated, self-degradation process that protects normal cells, but also promotes tumor cell survival under stress (11). Evidence suggests that endogenous HMGB1 is a critical regulator of autophagy in tumor cells. Cytoplasmic HMGB1 can directly bind to autophagy protein Beclin-1, disrupting Beclin-1/Bcl-2 interaction and sustaining autophagy (12). Endogenous knockdown of HMGB1 with siRNA or inhibition of its release with small-molecule inhibitors, abolished the protective effect of autophagy and increased tumor cell sensitivity to several clinically useful agents (10,13). Although autophagy may regulate selective HMGB1 release in some tumor types, the mechanisms of its extracellular release from gastric cancer cells undergoing autophagy and activation of surface receptor-mediated intracellular signaling pathways are not well characterized.

Multiple receptor types have been implicated in HMGB1 signaling, including the receptor for advanced glycation end products (RAGE) and members of the Toll-like family of receptors (TLRs) (14,15). In the present study, we showed that HMGB1 was highly expressed in human gastric carcinoma and primarily located in the cytoplasm of gastric cancer cells. Gefitinib, a common chemotherapeutic agent, activated autophagy and promoted the release of HMGB1, which was required to maintain gastric tumor cell survival. Intriguingly, the interaction between HMGB1 and RAGE initiated signaling involving ERK1/2 phosphorylation and contributed to the cell proliferation in gastric tumor cells.

## Materials and methods

### Patients and specimens

Gastric cancer samples and tissues surrounding the tumor (>2 cm from the tumor edge) were obtained from 23 patients that underwent curative gastric cancer resection at the Department of General Surgery in the First Affiliated Hospital of Fujian Medical University, China. Isolated tumors were only single gastric neoplasms; no patient received antitumor treatment before surgery. Specimens were prepared and maintained anonymously according to ethical and legal standards.

### Cell culture and reagents

We used the subsequent cell lines: BGC-823 (low differentiated stomach adenocarcinoma cell line), SGC-7901 (moderately differentiated stomach adenocarcinoma cell line) and gastric epithelial cells (GES-1) (immortalized fetal gastric mucosal cell line), MKN28 (well-differentiated adenocarcinoma cell line) and MKN45 (poorly differentiated adenocarcinoma cell line). These cell lines were kindly provided by Dr Yujuan Dong (Department of Surgery, The Chinese University of Hong Kong, Shatin, Hong Kong, China). The cell lines were routinely cultured in RPMI-1640 medium supplemented with 10% fetal bovine serum (FBS) (both from Gibco, Carlsbad, CA, USA) and maintained at 37°C in a humidified environment with 5% CO_2_. Gefitinib was obtained from Cayman Chemical (Ann Arbor, MI, USA) and glycyrrhizic acid, MTT, human recombinant HMGB1, rabbit-derived anti-human HMGB1 antibodies were obtained from Sigma. Neutralizing anti-RAGE, anti-TLR2 and anti-TLR4 and isotype-matched control (IgG) were from R&D Systems (Minneapolis, MN, USA), and the GFP-LC3 expression vector was kindly provided by Professor George G. Chen (Department of Surgery, The Chinese University of Hong Kong, Shatin, Hong Kong, China).

### Immunohistochemical analysis of HMGB1 expression in gastric cancer tissues

Human gastric cancer tissue sections were immunolabeled with rabbit anti-human HMGB1 antibodies (1:1,000) using a mouse/rabbit specific horseradish peroxidase (HRP)/diaminobenzidine (DAB) detection IHC kit (Abcam, San Francisco, CA, USA). Sections were incubated with biotinylated goat anti-rabbit antibodies and ExtrAvidin-conjugated HRP. Staining was developed with DAB chromogenic substrate and sections were counterstained with hematoxylin.

### Concentration of HMGB1 in serum and cell supernatant by ELISA

Serum from patients with gastric cancer and healthy volunteers was assayed. BGC-823 cells were treated with different doses of gefitinib for 24 h or with 20 μM gefitinib for designated time periods and the supernatant was collected for HMGB1 detection. The HMGB1 levels in serum and cell medium were quantified using an ELISA kit (Chemicon, Temecula, CA, USA) according to the manufacturer’s instructions.

### Immunofluorescence detection of HMGB1 expression in gastric cancer cells

Cells cultured on coverslips were fixated and processed with primary antibodies (anti-HMGB1, 1:500 dilution) followed by Alexa Fluor 488 conjugated anti-rabbit IgG (Invitrogen, Carlsbad, CA, USA). Nuclei were stained with DAPI (Invitrogen). Images were captured with a fluorescence microscope (Olympus IX81, Olympus, Tokyo, Japan).

### Western blot analysis

Lysates were isolated from the whole tissue homogenates or gastric cancer cells using a Total Protein Extraction kit (Millipore, Billerica, MA, USA) and were cytoplasmic and nuclear protein extracted using a Cytoplasmic and Nuclear Protein Extraction kit (Promega, Madison, WI, USA) and were subjected to western blot analysis. Antibodies against HMGB1 (1:8,000; Sigma), LC3B, ERK1/2, phospho-ERK1/2, p38, p38, AKT, phospho-AKT, JNK, phospho-JNK (1:1,000; Cell Signaling Technology, Danvers, MA, USA), RAGE (1:200; R&D Systems), β-tubulin, albumin and lamin B (1:1,000; Santa Cruz Biotechnology Inc., Santa Cruz, CA, USA) were used to develop immunoreactive signals. Densitometry was performed using AlphaImager 2200 system and Quantity software.

### GFP-LC3 analysis

BGC-823 cells were grown on coverslips and transfected with the GFP-LC3 vector using X-tremeGENE HP DNA (Roche Applied Science). Twenty hours later, the cells were treated with the selected agents for 24 h. Autophagic vesicles were monitored by GFP-LC3 aggregation in stably expressing polyclonal cell lines. A percentage of the cells with >10 GFP-LC3 puncta/100 cells from the two experiments were investigated using a Laser Scanning Confocal Microscope (Leica TCS SP5; Leica, Mannheim, Germany).

### Culture medium preparation

BGC-823 cells (2×10^6^) were cultured in a medium containing 20 μM gefitinib or DMSO for 24 h. The culture media were collected and used in a mixture with fresh media to treat BGC-823 cells for indicated periods. Cell proliferation was assessed via MTT assay.

### Cell growth assessment

Cell proliferation was analyzed with an MTT assay. Cells incubated with a medium containing different concentrations of HMGB1 or a mixture of cultured media were treated with 20 μl MTT dye (5 mg/ml) at 24, 48, 72 and 96 h. Optical density was determined at 570 and 630 nm using ELISA (Bio-Tek Instruments, Inc., Winooski, VT, USA). For the colony formation assay, BGC-823 colonies that contained >50 cells were counted and stained with 0.1% of crystal violet.

### RNA interference (siRNA)

RAGE siRNA (Shanghai GenePharma Co., Ltd., Shanghai, China) was transfected into the cells using X-tremeGENE siRNA (Roche Applied Science) according to the manufacturer’s protocol.

### Statistical analysis

All the results are expressed as mean ± SEM. Differences between the two groups were analyzed using a Student’s t-test and among multiple groups by a one-way analysis of variance with a Dunnett’s multiple comparison post hoc test. A two-way ANOVA followed by Dunnett’s test was performed for multiple comparisons. P<0.05 was considered statistically significant. Statistical analyses were performed using GraphPad Prism 6.0 (GraphPad Software, San Diego, CA, USA).

## Results

### HMGB1 is overexpressed in gastric cancer

**Figure 1:**
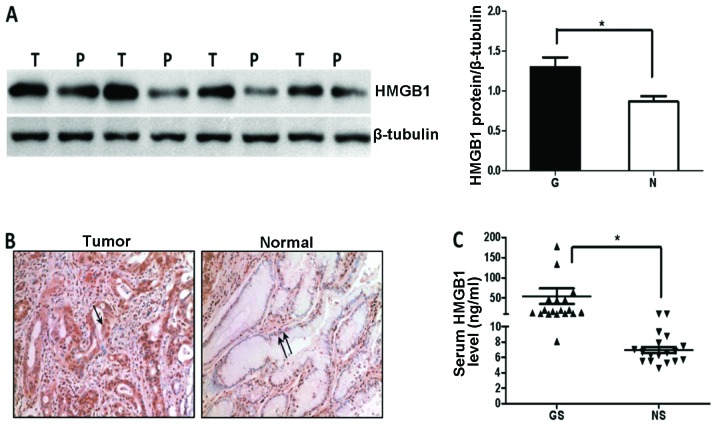
Expression of HMGB1 in gastric cancer tissues and serum. (A) HMGB1 protein levels were determined by western blot analysis in the non-tumorous and tumor tissues. Results were normalized to β-tubulin. Each value represents the average of 8 cases (^*^P=0.0101). (B) Immunohistochemistry of paraffin-embedded gastric tumor (n=30) and peritumor samples (n=23). HMGB1 in cytoplasm (arrows) and in the nucleus (double arrows) (x400 magnification). (C) HMGB1 expression in serum of gastric cancer patients (n=19) and healthy controls (n=18) was detected by ELISA, expressed as the mean ± SEM. Each triangle represents an individual patient; bars indicate the mean (^*^P=0.023). HMGB1, high mobility group box-B1. GS, gastric cancer serum; NS, normal serum from healthy individuals.

We first examined the amount of HMGB1 in 8 gastric cancer tissue samples and corresponding non-tumor gastric tissues by immunoblotting analysis. Expression of HMGB1 protein was significantly higher in the tumor compared to that in the peritumor tissues (P=0.0101, Fig. 1A). HMGB1 was predominantly localized in the tumor cell cytoplasm, while low expression was detected mainly in the peritumor cell nuclei (Fig. 1B). HMGB1 was actively released into the circulation of patients with gastric cancer and serum levels were significantly increased in gastric cancer patients compared to the level in the healthy volunteers (Fig. 1C).

### Overexpression and cytoplasmic localization of HMGB1 in gastric cancer cells

**Figure 2:**
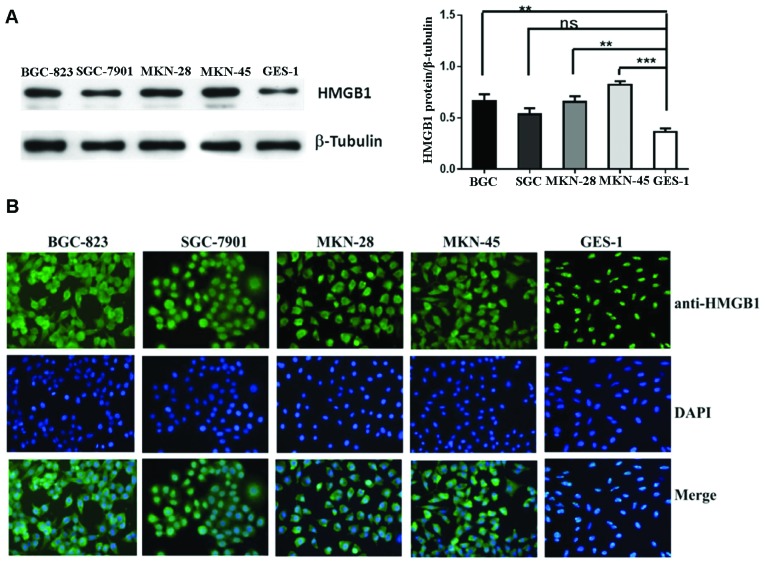
Overexpression and localization of HMGB1 in gastric cancer cells. (A) HMGB1 expression in four gastric cancer cell lines and non-malignant gastric epithelial GES-1 cells. Protein expression was normalized to β-tubulin (right graph). Each value represents the average of three independent experiments. (^**^P<0.01 and ^***^P <0.001; ns, not significant). (B) Nuclear and cytoplasmic HMGB1 localization visualized by immunostaining with the anti-HMGB1 antibody (green) and DAPI (blue). Image shown is representative of three experiments (x400 magnification). HMGB1, high mobility group box-B1; GES-1, gastric epithelial cell line.

Western blot analysis and immunofluorescence were performed in four gastric cancer cell lines and non-malignant GES-1 cells and showed that HMGB1 protein expression was much higher in the cancer cells compared to the level in the GES-1 cells (Fig. 2). HMGB1 was absent in the cytoplasm of the non-malignant gastric epithelial cells, whereas cytoplasmic HMGB1 was abundant in all four cancer cell lines. High extracellular and cytoplasmic levels of HMGB1 suggest that its detection may occur in the context of active HMGB1 release.

### Autophagy induces HMGB1 release from gastric cancer cells

**Figure 3:**
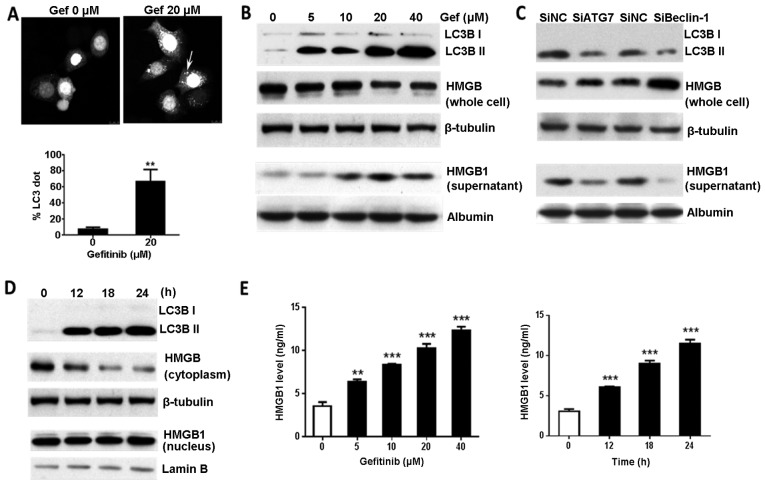
Release of HMGB1 from autophagic gastric cancer cells induced by chemotherapeutic agent. (A) GFP-LC3-transfected BGC-823 cells 24 h following gefitinib (Gef) or control treatment. White arrow indicates the characteristic punctate pattern of GFP-LC3. (B) Western blot analysis of intracellular and extracellular (supernatant) HMGB1 and LC3 in whole cell lysates and supernatants from the BGC-823 cells treated with gefitinib (Gef) (0, 5, 10, 20 and 40 μM) for 24 h. (C) BGC-823 cells transfected with control or ATG7 and Beclin-1 siRNA for 24 h were treated with gefitinib (20 μM) for a further 24 h. Culture medium and whole cell lysates were subjected to western blot analysis. (D) Cytoplasmic and nucleic extracts from BGC-823 cells subjected to a time course of gefitinib (20 μM). HMGB1 and LC3 were determined by western blot analysis. β-tubulin, Lamin B and albumin served as loading controls. Blots shown are representative of three experiments. (E) BGC-823 cells were treated with various concentrations of gefitinib (left) or 20 μM gefitinib for the indicated time periods (right), HMGB1 in cultured medium was quantitated by ELISA. Assays were conducted in triplicate for each experiment of three independent experiments. Multiple comparisons were performed by one-way ANOVA followed by Dunnett’s test (^**^P<0.01 and ^***^P<0.001 vs. control). HMGB1, high mobility group box-B1.

HMGB1 is released from tumor cells undergoing classical necrotic cell death. Recent discoveries suggest that autophagy regulates selective HMGB1 release in tumor cells (8,16). In the present study, we observed that gefitinib, an epidermal growth factor receptor (EGFR) inhibitor, activated autophagy in the gastric cancer cells, as indicated by LC3-positive puncta and increased the levels of the autophagosome-bound form of LC3, LC3 II (Fig. 3A and B). Gefitinib-induced autophagy triggered a dose-dependent increase in HMGB1 release into the media of the BGC-823 cells. Western blot analysis showed that HMGB1 protein was reduced in the BGC-823 cells and increased in the supernatants, after treatment with gefitinib (Fig. 3B). Knockdown of Atg7 or Beclin-1 prevented HMGB1 release from the gefitinib-treated cells suggesting regulation by autophagy (Fig. 3C). At 12 h post-gefitinib treatment, cytoplasmic HMGB1 levels declined, however, nuclear HMGB1 expression showed no significant change by 24 h after treatment (Fig. 3D). Furthermore, we utilized ELISA to quantify the HMGB1 released in culture supernatants. Consistent with the western blot analysis results, the HMGB1 released from the gefitinib-treated cells was significantly increased in a time-and dose-dependent manner compared with that released in the untreated cells (Fig. 3E). This demonstrated that HMGB1, particularly the cytoplasmic HMGB1, was rapidly released and accumulated in the culture media of the gefitinib-treated gastric tumor cells.

### Effect of extracellular HMGB1 on gastric cancer cell proliferation

**Figure 4:**
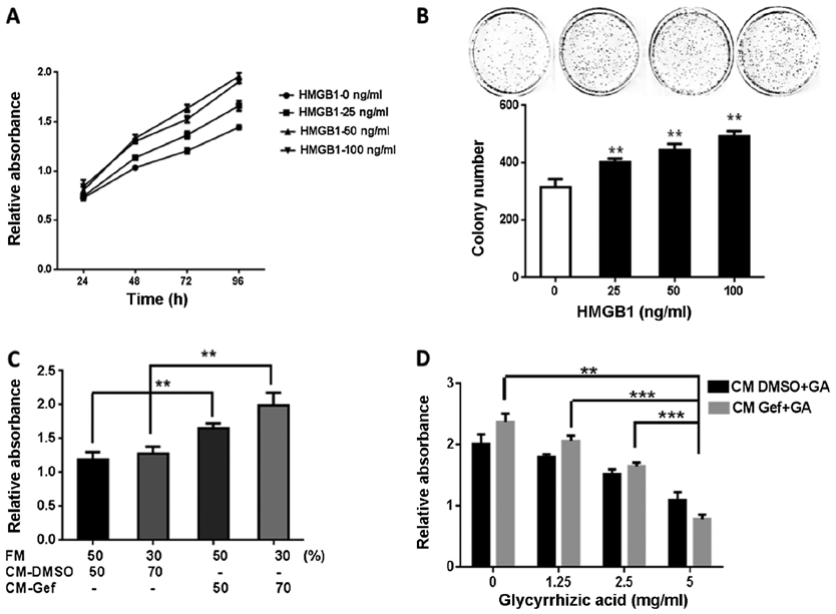
Effects of extracellular HMGB1 on gastric cancer cell proliferation. (A) MTT assay was used to determine the cell growth curves in the BGC-823 cells treated for four days with the indicated concentrations of human recombinant HMGB1. (B) Colony formation was assayed in cells cultured with human recombinant HMGB1 (0, 25, 50 and 100 ng/ml) for 10 days. Colonies were stained, counted and photographed. Average cell numbers/colony in single-cell colony forming assay. (C) BGC-823 cells were incubated for 24 h with medium from BGC-823 cells treated with 20 μM gefitinib or DMSO, mixed with fresh medium. FM, fresh medium; CM-DMSO, culture medium of DMSO-treated cells; CM-Gef, culture medium of 20 μM gefitinib-treated cells. (D) BGC-823 cells were incubated for 24 h with CM-Gef medium containing various doses of glycyrrhizic acid. Cell proliferation was determined via MTT assay in three separate experiments, each performed in quadruplicate. Differences were analyzed using a Student’s t-test between two groups or one-way analysis of variance ANOVA with Dunnett’s test among multiple groups (^**^P<0.01 and ^***^P<0.001 vs. the control group). HMGB1, high mobility group box-B1.

The extracellular HMGB1 effects on cell proliferation and growth were examined via MTT and colony formation assays in BGC-823 cells. Human recombinant HMGB1 significantly enhanced the cell proliferation in a dose- and time-dependent manner compared to that in the control group (Fig. 4A). The colony formation rate of the BGC-823 cells was higher in the HMGB1-treated vs. the control group (Fig. 4B). The proliferative effect of the natural HMGB1 released by autophagy was assessed in the BGC-823 cells cultivated with a gefitinib-treated cell medium, with or without glycyrrhizic acid, a known inhibitor of HMGB1 (17). Cells cultivated with gefitinib-treated cell medium demonstrated an enhanced growth compared to the DMSO-treated controls (Fig. 4C and D). Glycyrrhizic acid noticeably suppressed the growth promoting-effect of gefitinib (Fig. 4D). These findings indicated that glycyrrhizic acid bound to HMGB1, released by cells undergoing autophagy and attenuated HMGB1-induced tumor proliferation.

### HMGB1 induces ERK activation in gastric tumor cells

**Figure 5:**
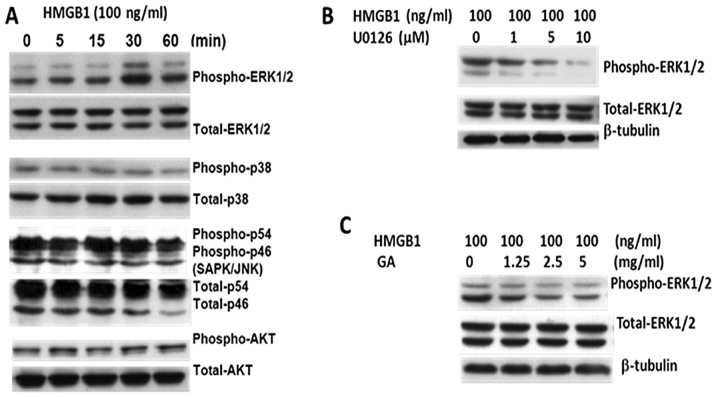
Extracellular HMGB1 increases ERK1/2 phosphorylation in gastric cancer cells. (A) Twenty-four hour serum-starved BGC-823 cells were subjected to 100 ng/ml HMGB1 for the indicated time period, and western blot analysis was performed for the phosphorylated forms of p-ERK1/2 (Thr202/Tyr204), p38 (Thr180/Tyr182), p-JNK (Thr183/Tyr185), p-AKT (Thr308) and their corresponding non-phosphorylated proteins. (B) Starved BGC-823 cells were pretreated with the MAPK inhibitor U0126 or the media (control) for 1 h and then treated with 100 ng/ml of HMGB1 for 30 min and subsequently phosphorylated and total ERK1/2 were detected by western blot analysis. (C) Cell lysates from the starved BGC-823 cells were treated with HMGB1 (100 ng/ml) with or without glycyrrhizic acid (GA) for 30 min and analyzed by anti-phospho-ERK1/2 (p-ERK1/2) antibodies. Data are from one of the three separate experiments. HMGB1, high mobility group box-B1; MAPK, mitogen-activated protein kinase.

HMGB1 activates multiple signaling pathways involved in cell proliferation, including mitogen-activated protein kinase (MAPK), AKT and JNK pathways (14,18,19). BGC-823 cells were incubated with human recombinant HMGB1 for different times and harvested for analysis of ERK1/2 phosphorylation via western blot analysis. HMGB1 induced ERK activation in a time-dependent manner (Fig. 5A); however, p38 MAPK, AKT and JNK phosphorylation and activation were not detected (Fig. 5A). Treatment with U0126 (MEK1 and MEK2-specific inhibitor) and glycyrrhizic acid significantly reduced the ERK1/2 phosphorylation (Fig. 5B and C). Therefore, HMGB1 induced cell proliferation in gastric cancer cells via activation of the MEK/ERK signaling pathway.

### RAGE is required for HMGB1-induced MAPK activation and gastric cancer cell proliferation

**Figure 6:**
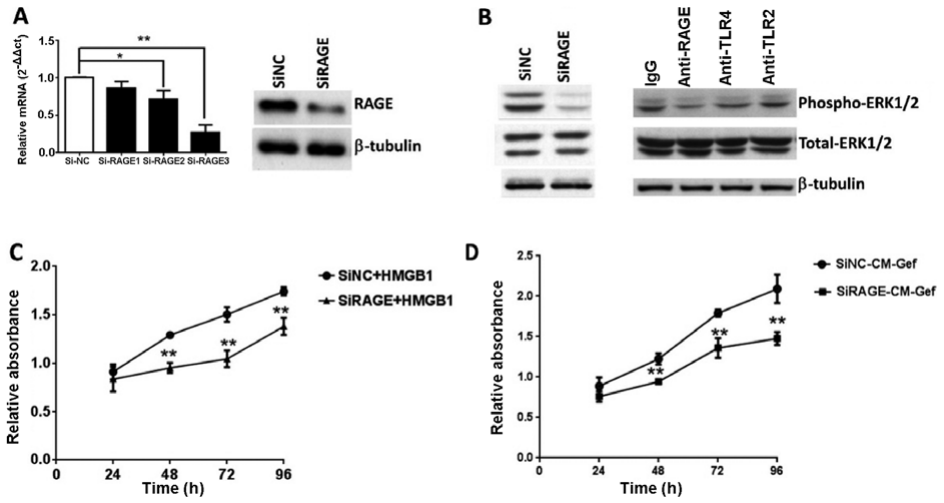
Targeted knockdown of RAGE results in inhibition of ERK activation and decreases cell proliferation following treatment with HMGB1 or mixed medium. (A) BGC-823 cells were transfected with 40 pmol siRAGE and scrambled non-silencing siRNA (siNC). Knockdown efficiency of siRAGE was assessed by real-time PCR and western blot analysis; data are represented as RAGE transcript levels normalized to siNC (set to 1). Experiments were repeated 3 times in triplicate; ^*^P<0.05 and ^**^P<0.01. (B) RAGE-reduced or control BGC-823 cells were treated with HMGB1 (100 ng/ml) and harvested to detect ERK1/2 activation (left). BGC-823 cells were pretreated with anti-RAGE antibodies (10 μg/ml), anti-TLR4 (50 ng/ml), anti-TLR2 (50 ng/ml) and IgG, and subsequently subjected to HMGB1 (100 ng/ml) for 30 min (right). Active and total ERK were detected by western blot analysis. (C) siRAGE and siNC were transfected into the BGC-823 cells and 24 h later the cells were treated with 100 ng/ml HMGB1 for 96 h. (D) The transfected cells were incubated with a mixed medium for 96 h. Proliferation was determined via MTT assay in 4 separate experiments, each performed in quadruplicate. Differences between siRAGE and siNC groups at different time-points were analyzed using two-way analysis of variance ANOVA with Dunnett’s test (^**^P<0.01 compared to the siNC group). HMGB1, high mobility group box-B1.

Several receptors have been linked to HMGB1 signaling, including RAGEs and TLRs (14,15). We analyzed TLR2, TLR4 and RAGE gene expression in human gastric tumor cells using quantitative real-time PCR. The three receptors were expressed in multiple gastric tumor cell lines *in vitro*. Interestingly, RAGE expression was greater in the BGC-823 cells, where HMGB1 was relatively highly expressed (data not shown). To assess the contribution of RAGE to ERK activation and tumor growth, siRNA was designed to silence RAGE. RAGE expression was significantly reduced in the siRAGE group as long as 96 h post-transfection, which covered the maximal duration of the cell proliferation assay (Fig. 6A). RAGE knockdown almost completely abrogated ERK phosphorylation in response to HMGB1 (Fig. 6B). To confirm the interaction of extracellular HMGB1 and its receptor, anti-TLR2, anti-TLR4 and anti-RAGE antibodies were used to block the respective receptors in BGC-823 cells. Consistent with the RAGE knockdown results, anti-RAGE antibodies significantly lowered the ERK response to HMGB1, although anti-TLR2 and anti-TLR4 antibodies demonstrated no inhibition of ERK response (Fig. 6B). Thus, HMGB1-induced ERK activation is dependent on RAGE. Cells subjected to different treatments were analyzed via MTT assay to investigate the RAGE involvement in HMGB1-induced cell proliferation. Compared to the siRNA-control, the RAGE-reduced BGC-823 cells displayed markedly decreased proliferation in response to recombinant HMGB1 or mixed medium (a mixture of gefitinib-treated cell medium and fresh medium in a ratio of 7:3) (Fig. 6C and D). Extracellular HMGB1 interacted with RAGE and activated ERK signaling responsible for gastric cancer cell proliferation.

## Discussion

Increased HMGB1 is observed in many cancer types, including prostate cancers, leukemia, colorectal and hepatocellular cancer and is related to occurrence, progression, and metastasis (10,20–22). HMGB1 expression has also been described in gastric epithelial cancer (1–3). Nearly all gastric adenocarcinomas show HMGB1-positive labeling, primarily in the nucleus and HMGB1 in gastric cancer cells may be significantly increased compared to that in the epithelial and stromal cells in normal tissues (1). Chung *et al* (2) reported that the serum HMGB1 levels were higher than normal in patients with gastric cancer, while a positive correlation was observed between serum levels and the depth of invasion, lymph node metastasis, tumor size and poor prognosis. Similar results were obtained in the present study. We observed that the HMGB1 expression in gastric cancer tissues was increased compared to the non-cancerous tissues, while the serum HMGB1 levels in cancer patients were higher than that in the healthy volunteers (Fig. 1). Gastric carcinoma cell lines (BGC-823, SGC-7901, MKN-28 and MKN-45) exhibited high HMGB1 levels in both the nuclei and cytoplasm, whereas gastric epithelial cells showed a reduced HMGB1 level, primarily localized to the nucleus (Fig. 2). High serum HMGB1 levels in cancer patients and predominant cytoplasmic localization indicate that HMGB1 can be actively released into the circulation.

HMGB1 is actively secreted from activated innate immune cells or passively from cells undergoing classical necrotic cell death (4). Recently, it was observed that HMGB1 was selectively released from tumor cells undergoing autophagy (8,16). Evidence suggests that HMGB1 may induce autophagy in cancers associated with increased sensitivity to cytotoxic anticancer agents (10). Contrarily, HMGB1-mediated autophagy may protect gastric cancer cells from the chemotherapeutic vinca alkaloid, vincristine (23). In the present study, data indicated that the protective effects of HMGB1 occurred through its upregulation of the protein myeloid cell leukemia-1 (Mcl-1). Other studies suggest that vincristine may reduce Mcl-1 expression and promote the death of cancer cells (24), complicating the interpretation of our findings. Autophagy, a process in which subcellular membranes undergo dynamic morphological changes resulting in intracellular degradation of proteins, cytoplasmic organelles and pathogens, is a mechanism exploited by tumor cells for survival and used in determining tumor response to anticancer therapy. Increasing evidence suggests that autophagy represents a resistant mechanism to chemotherapy in many malignancies and our findings support this notion. Here we observed that BGC-823 cells (an EGFR-rich human gastric carcinoma cell line) were resistant to the EGFR tyrosine kinase inhibitor, gefitinib. The IC_50_ value of gefitinib for growth inhibition of BGC-823 cells was 92.83±1.92 μM (data not shown).

To investigate the effect of gefitinib on autophagy, we employed transiently expressed GFP-LC3 in BGC-823 cells and quantified puncta formation. Gefitinib (20 μM) increased autophagic flux (Fig. 3A) and increased autophagosome-bound LC3II in the BGC-823 cells in a dose- and time-dependent manner (Fig. 3B and D). We then investigated the HMGB1 levels in the BGC-823 cell supernatants and homogenates after treatment with low doses of gefitinib (10, 20 and 40 μM), which did not affect the cell viability. HMGB1 accumulated rapidly in the culture medium and was slightly reduced in the homogenates after adding gefitinib. The increased levels of extracellular HMGB1 in the gefitinib-treated supernatants were confirmed by ELISA. Unlike dying cells, no significant release of lactate dehydrogenase (LDH) was detected in the autophagic cells induced by gefitinib (data not shown). Moreover, gefitinib-induced HMGB1 release was prevented by knockdown of Atg7 or Beclin-1 in the BGC-823 cells (Fig. 3C), which confirmed HMGB1 release due to increased autophagy. HMGB1 levels in the cytoplasm and nucleus were assessed at different time-points following gefitinib treatment and cytoplasmic levels were decreased at 12 h post-treatment. However, HMGB1 levels did not change after the DMSO treatment (Fig. 3D). These data show that HMGB1, particularly the cytoplasmic HMGB1, is rapidly released and accumulated in the culture media after gefitinib-induced autophagy in gastric cancer cells.

Autophagy is a cell survival mechanism in many types of malignant tumors (25,26). Kang *et al* (27) found that intracellular HMGB1 regulates autophagy through interaction with Beclin-1, in competition with Bcl-2, indicating its functional importance in cross-regulating apoptosis and survival. However, detailed functions of extracellular HMGB1 remain largely undefined. Our data showed that exogenous HMGB1 induced a dose-dependent upregulation of BGC-823 cell proliferation (Fig. 4A and B). Treatment with a medium containing HMGB1 released from the autophagic cells resulted in enhanced cell growth. Therefore, HMGB1 released by autophagic cells serves as a pro-survival signal for residual cells.

HMGB1 acts as a growth factor for cancer cells, it activates MAPK or PI3K/AKT signaling and enhances proliferation via RAGE receptor activation (18,28,29). Our findings confirmed that exogenous HMGB1 increased ERK1/2 phosphorylation, with no effect on the phosphorylation of p38, JNK and PI3K/AKT pathways in BGC-823 cells (Fig. 5A). HMGB1-induced ERK1/2 activation was blocked by pretreatment with either U0126, an MEK1/2 inhibitor, or glycyrrhizic acid, an HMGB1 inhibitor. Therefore, we propose that extracellular HMGB1 regulates cell proliferation through MEK-ERK signaling. Recently, numerous studies indicated that RAGE, a multi-ligand receptor for certain stress-associated factors, affected the proliferation of various types of cancer cells (14,29,30). Consequently, we employed siRNA to silence RAGE and investigate its function in our experimental model (Fig. 6A). ERK activation was partially reversed in the RAGE-reduced BGC-823 cells (Fig. 6B). Anti-RAGE antibody, but not anti-TLR2 and anti-TLR4 antibodies, significantly inhibited the ERK response to HMGB1 (Fig. 6B). RAGE knockdown also suppressed HMGB1-induced cell proliferation validating our presumption that HMGB1/RAGE interaction modulates gastric cancer cell proliferation. Therefore, we deduce that exogenous HMGB1 binds to RAGE and initiates MEK/ERK signal transduction, a process that may play a crucial role in cancer cell survival and resistance to chemotherapy.

**Figure 7:**
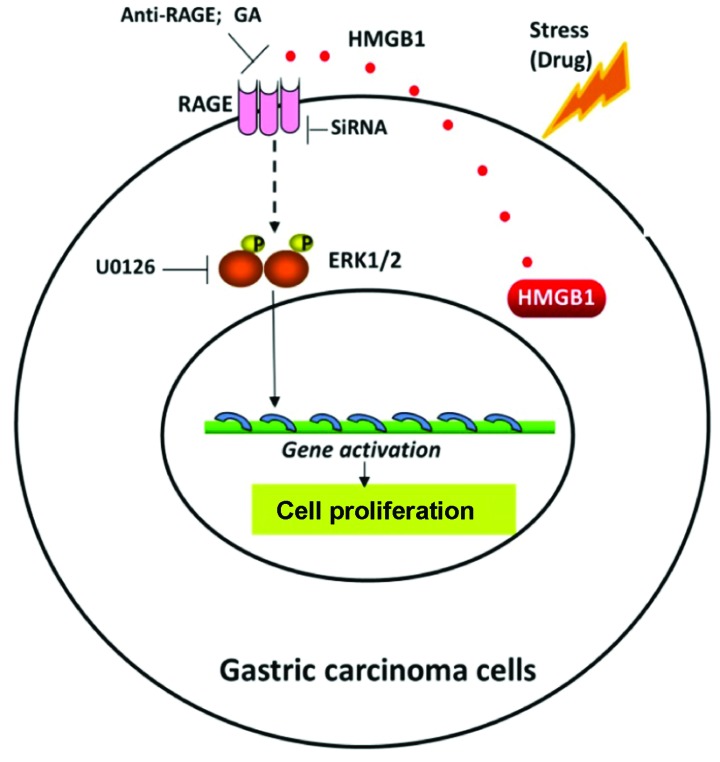
Model of the extracellular release of HMGB1 and regulation of cell proliferation during chemotherapy. In gastric cancer cells, HMGB1 is released following chemotherapy-induced protective autophagy in cells, as a damage-associated molecular pattern molecule. Increased HMGB1 in the tumor microenvironment binds primarily to RAGE, which initiates increased phosphorylation of ERK1/2 (p-ERK1/2) and induces cell proliferation, leading to cancer regrowth and drug resistance. HMGB1, high mobility group box-B1.

In conclusion, the present study demonstrated (Fig. 7) that HMGB1 was highly expressed, particularly within the cytoplasm of human gastric carcinoma cells. As a regulator of cell death and survival, HMGB1 was released from the tumor cells undergoing gefitinib-induced autophagy, bound with RAGE and initiated signaling involving phosphorylation of ERK1/2, which contributed to gastric tumor cell proliferation. Thus, we propose HMGB1 release as a pro-survival signal for residual cells following various cytotoxic cancer treatments. HMGB1 inhibitors or RAGE suppressants may be effective in prohibiting cancer regrowth, supported by HMGB1-related autophagy during chemotherapy. Such methods may be considered for future chemotherapy protocols to increase their efficacy in human gastric adenocarcinoma and other epithelial neoplasms.
